# Lung elastance and PEEP level with lowest transpulmonary driving pressure can be determined by a rapid PEEP step procedure without esophageal pressure measurements

**DOI:** 10.1186/s13054-023-04590-8

**Published:** 2023-08-03

**Authors:** O. Stenqvist

**Affiliations:** Sahlgrenska Academy, Gothenburg University, Gothenburg, Sweden

In a recent study by Mojoli et al. [1], tidal lung hysteresis is suggested as a way of interpreting PEEP induced changes in compliance in ARDS patients. The measurement procedure comprises a recruitment manoeuvre and a PEEP step down trial from 6 cm H_2_O above, to 6 cm H_2_O below clinical PEEP with a slow inflation-deflation to an airway plateau pressure of 30 cm H_2_O at each PEEP level. The first figure of the Mojoli study presents three typical patterns of respiratory system compliance (CRS) and tidal hysteresis volume—high, biphasic and low tidal recruitability.

It has been shown that the change in end-expiratory lung volume (ΔEELV) is determined by the size of the PEEP step and *lung* elastance as ΔPEEP/EL [2]. This is explained by the balance at functional residual capacity (FRC) between the elastic recoil of the lung on one hand and the rib cage spring out force (RCSOF), which strives to its resting volume at 70–80% of total lung capacity (TLC), on the other hand. Thus, FRC is the highest lung volume the RCSOF can achieve. If a pneumothorax is implemented, i.e., exterior lung and interior thoracic cavity surface are disconnected, the thoracic cavity will expand to its resting volume. This implies that if the end-expiratory lung volume is increased above FRC by PEEP, ΔPEEP only has to overcome the elastic recoil of the lung, while the RCSOF expands the chest wall complex (chest wall and diaphragm) and consequently, transpulmonary pressure increases as much as PEEP is increased.

Therefore, if ΔEELV is determined by the ventilator pneumotachograph, lung elastance can be determined as ΔPEEP/ΔEELV, without esophageal pressure measurements. In the first figure of the Mojoli, the end-expiratory airway pressure/volume points can be identified in three typical patients and therefore, the lung pressure/volume curve determined by the PEEP step method from end-expiration at the lowest PEEP level to end-inspiration at the highest PEEP level of the measurement procedure [2]. In addition, tidal respiratory system elastance (ERS) and the calculated the ratio of EL/ERS at each PEEP level were calculated (Fig. 1) (for details, see Additional file 1).

**Fig. 1:**
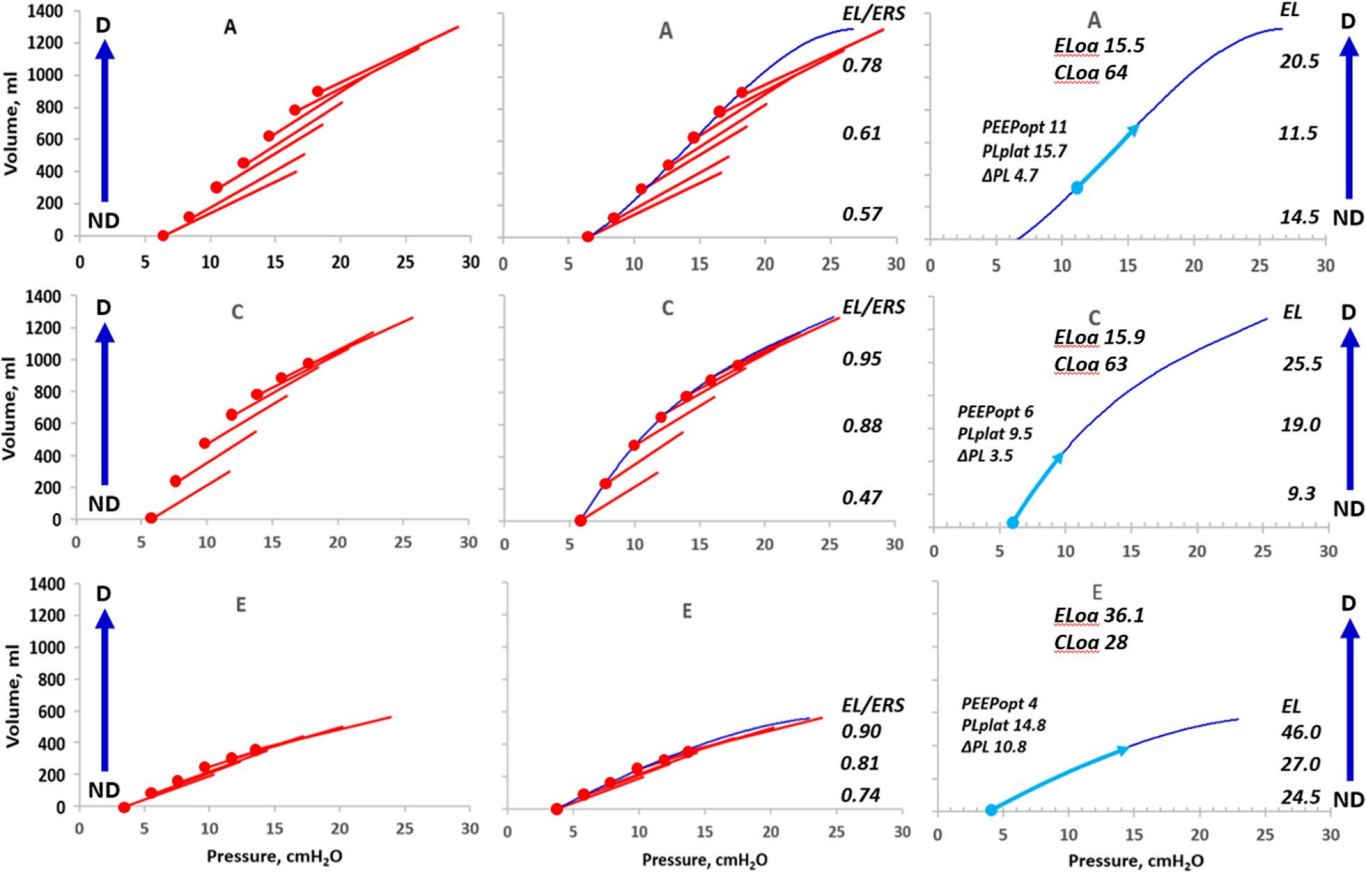
Left panels: Tidal airway P/V curves (red) of patient A (400 ml), C (300 ml) and E (200 ml) of the Mojoli study. Mid panels: Tidal airway PV curves with lung P/V curve (blue line) through end-expiratory airway P/V points (red dots). Right panels: Lung P/V curves (light blue arrows) of 400 ml tidal volume at the PEEP level where transpulmonary driving pressure (ΔPL) is lowest and thus, least injurious, optimal PEEP (PEEPopt). PLplat is the end-inspiratory transpulmonary plateau pressure of the 400 ml tidal volume, which corresponds to a tidal volume of 6 ml/kg in a person with 70 kg ideal body weight (ibw). The dark blue vertical arrows indicate that PEEP inflation starts in non-dependent lung region (ND) and proceeds towards dependent regions (D) [3]. Mid panels show that in patient A and C, EL/ERS was low at the lowest PEEP level but increased at the highest PEEP level, as seen in extrapulmonary ARDS. In the E patient, EL/ERS was high already at the lowest PEEP level and increased further with increasing PEEP, which indicates a low impact of the chest wall complex on respiratory system mechanics, i.e., a behaviour seen in pulmonary ARDS. The overall lung elastance (ELoa) was 15.5, 15.9 and 36.1 cm H_2_O/L in patient A, C and E, respectively, (corresponding to overall lung compliance of 64, 63 and 28 ml/cm H_2_O). In patient A, the lung P/V curve has a classic sigmoid appearance and tidal volume is occurring at more dependent lung regions with increasing PEEP. In patient C, the tidal volume is not transferred towards dependent direction with increasing PEEP. Instead, lung elastance increases from a very high level PEEP step by PEEP step. This indicates a true “baby” lung positioned in the most non-dependent lung region above a more or less consolidated dependent lung. The C patient has a pattern that is similar to patient E but with much higher volumes inflated. One could speculate that this is a patient with mild/moderate emphysema on top of a consolidated lung

A safe airway driving pressure (ΔPAW) should be below 15 cm H_2_O [4], which corresponds to a transpulmonary driving pressure (ΔPL) below 10 cm H_2_O, as average ratio of lung to respiratory system elastance, EL/ERS, is ≈ 0.70 [2]. In patient A and C, application of a protective tidal volume of 400 ml (6 ml/kg ibw in a 70 kg ibw patient) results in optimal PEEP levels of 11 and 6 cm H_2_O with safe ΔPL of 4.7 and 3.5 cm H_2_O, respectively. In patient E, ΔPAW is only 13 cm H_2_O, which is seemingly safe as it is well below the ΔPAW safety limit of 15 cm H_2_O. But, as lung elastance is extremely high, a 400 ml tidal volume results in a ΔPL of 10.8 cm H_2_O, *above the ΔPL* safety limit. This underlines the need for individualisation of tidal volume and PEEP setting according to *the mechanical properties of the lung and not according to the respiratory system.*

The Mojoli study introduces hysteresis as a new mode of interpreting PEEP induced CRS changes during a multi-PEEP step trial of more than 20 min duration. PEEP according to best CRS, tidal hysteresis and to the combination of the two, did not coincide. In addition, the relationship between hysteresis PEEP and ventilator induced lung injury is unknown.

However, we have developed a method where the PEEP level where the *transpulmonary* driving pressure is lowest (least injurious) can be determined by a rapid two-PEEP step procedure from a clinical PEEP of 5–8 cm H_2_O to 18–20 cm H_2_O, with a three minute duration [2, 5]. As the method is noninvasive, it is easy to implement during difficult clinical situations as during the covid pandemic. In addition, it is possible to use it directly at start of mechanical ventilation, when an appropriate PEEP setting may prevent further lung collapse and consolidation.

A semi-automatic software determines ΔEELV between the three PEEP levels as the cumulative difference in expiratory tidal volume, the lung P/V curve between the end-expiratory airway = lung P/V points and up to the end-inspiratory transpulmonary plateau pressure/volume point at the highest PEEP level. On this lung P/V curve, both end-expiratory and end-inspiratory transpulmonary P/V points are positioned, as the transpulmonary pressure at a certain lung volume is the same, irrespective of whether this volume level has been reached by tidal or PEEP inflation. Thus, from the equation for the lung P/V curve, not only the optimal PEEP level for the tidal volume used during the measurement procedure can be calculated, but also the transpulmonary driving pressure of any combination of PEEP and tidal volume, as any tidal lung P/V curve is positioned on the full lung P/V curve derived from the two-PEEP step measurement procedure.

### Supplementary Information

**Additional file 1**. e-supplement of Lung elastance and PEEP level with lowest transpulmonary driving pressure can be determined by a rapid PEEP step procedure without esophageal pressure measurements. Physiologic background. Determinants of DEELV. Analysis details.

## Data Availability

Not applicable.

## References

[1] Mojoli F, Pozzi M, Arisi E, Mongodi S, Orlando A, Maggio G, Capra Marzani F, Brochard L (2023). Tidal lung hysteresis to interpret PEEP-induced changes in compliance in ARDS patients. Crit Care.

[2] Grivans C, Stenqvist O (2022). Gas distribution by EIT during PEEP inflation: PEEP response and optimal PEEP with lowest trans-pulmonary driving pressure can be determined without esophageal pressure during a rapid PEEP trial in patients with acute respiratory failure. Physiol Measur.

[3] Gattinoni L, Pelosi P, Crotti S, Valenza F (1995). Effects of positive end-expiratory pressure on regional distribution of tidal volume and recruitment in adult respiratory distress syndrome. Am J Respir Crit Care Med.

[4] Amato MB, Meade MO, Slutsky AS, Brochard L, Costa EL, Schoenfeld DA, Stewart TE, Briel M, Talmor D, Mercat A (2015). Driving pressure and survival in the acute respiratory distress syndrome. N Engl J Med.

[5] Persson P, Stenqvist O (2022). Protective positive end-expiratory pressure and tidal volume adapted to lung compliance determined by a rapid positive end-expiratory pressure-step procedure in the operating theatre: a post hoc analysis. Br J Anaesth.

