# Supplementary material for: Lung elastance and PEEP level with lowest transpulmonary driving pressure can be determined by a rapid PEEP step procedure without esophageal pressure measurements
Source: Crit Care. 2023 Aug 3;27:306. doi: 10.1186/s13054-023-04590-8 (PMC10399046; doi:10.1186/s13054-023-04590-8)
Supplement: Supplementary file 1 — Additional file 1. e-supplement of Lung elastance and PEEP level with lowest transpulmonary driving pressure can be determined by a rapid PEEP step procedure without esophageal pressure measurements. Physiologic background. Determinants of DEELV. Analysis details. [file 13054_2023_4590_MOESM1_ESM.pdf]

E-supplement of

Lung elastance and PEEP level with lowest transpulmonary driving pressure can be determined by a rapid PEEP step procedure without esophageal pressure measurements

O. Stenqvist
Sahlgrenska Academy, Gothenburg University, Gothenburg, Sweden

ΔEELV is determined by lung elastance and the size of the PEEP step

As the chest wall complex does not lean on the lung at FRC, or at end-expiration at increased PEEP, ΔPEEP only has to overcome the elastic recoil of the lung, while the rib cage spring out force fends of the chest wall complex. Thus ΔEELV = ΔPEEP/EL (fig. 1).

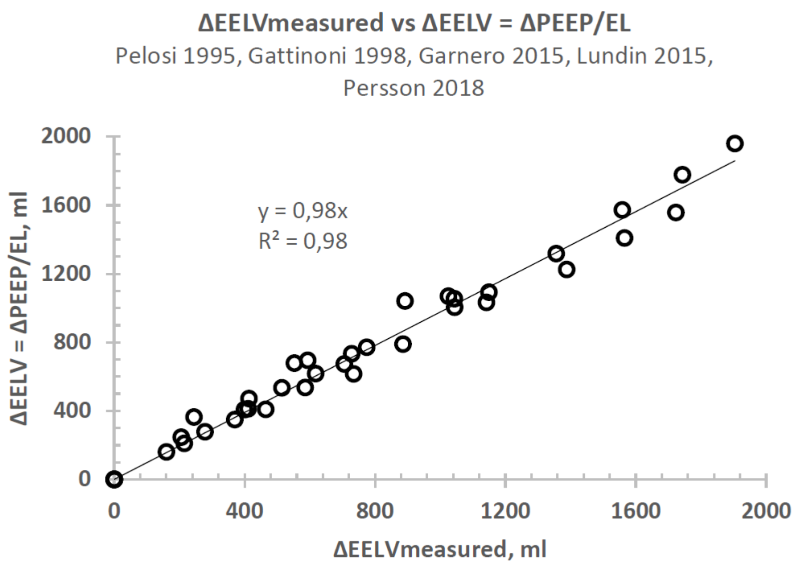

Fig. 1. Cumulative ΔEELV measured by the ventilator pneumotachograph compared to ΔEELV calculated as the change in PEEP divided by the lung elastance, ΔPEEP/ ΔL, where lung elastance was determined as the transpulmonary driving pressure divided by the tidal volume, using esophageal pressure measurements, ΔPL/VT. In the studies by Gattinoni et al [2] and Pelosi et al [1] PEEP steps were performed from ZEEP to respective PEEP level and back, 0-5-0, 0-10-0, 0-15-0 cmH$_2$O in random order. The increase in end-expiratory lung volume (ΔEELV) between two PEEP levels was measured as the difference in volume exhaled from end-inspiration at PEEP to ZEEP during a prolonged expiration. In the Garnero et al study [3] PEEP steps were performed consecutively, starting at a PEEP of 5 cmH$_2$O, 5-10-15-20-25-30-35-40 cmH$_2$O. In the Lundin [4], Garnero and Persson [5] studies, ΔEELV was measured as the cumulative difference in inspiratory and expiratory tidal volume between pressure/volume (P/V) equilibrium of two PEEP levels [6]. With permission from Intensive Care Medicine [7].

As ΔEELV is equal to ΔPEEP/EL,

EL = ΔPEEP/ΔEELV

and EL is also equal to transpulmonary driving pressure divided by tidal volume,

ΔPL/VT

Consequently, when ΔEELV = VT,

ΔPEEP = ΔPL ≈ ΔPLEE = ΔPL (Fig. 2)

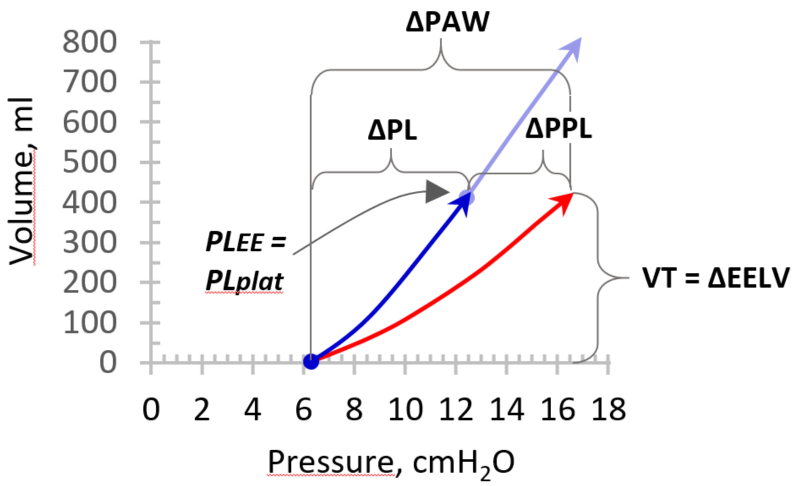

Fig. 2. Tidal airway (red arrow) and lung P/V curves (dark blue arrow) in patient A at PEEP 6 cmH_2O. The tidal lung P/V curve at PEEP 12 (light blue arrow) starts at the same pressure (PLEE) as the transpulmonary plateau pressure at PEEP 6, as VT ≈ ΔEELV. The difference between end inspiratory plateau pressure of the tidal volume from PEEP 6 cmH_2O and PEEP 12 cmH_2O (= $PLEE_{12}$ = $PLplat_6$) is equal to tidal pleural pressure variation, ΔPPL.

The implication of ΔPEEP = ΔPL when ΔEELV = VT, is that at a certain lung volume level, transpulmonary pressure is the same, irrespective of whether this lung volume level has been reached by tidal or PEEP inflation (Fig. 3).

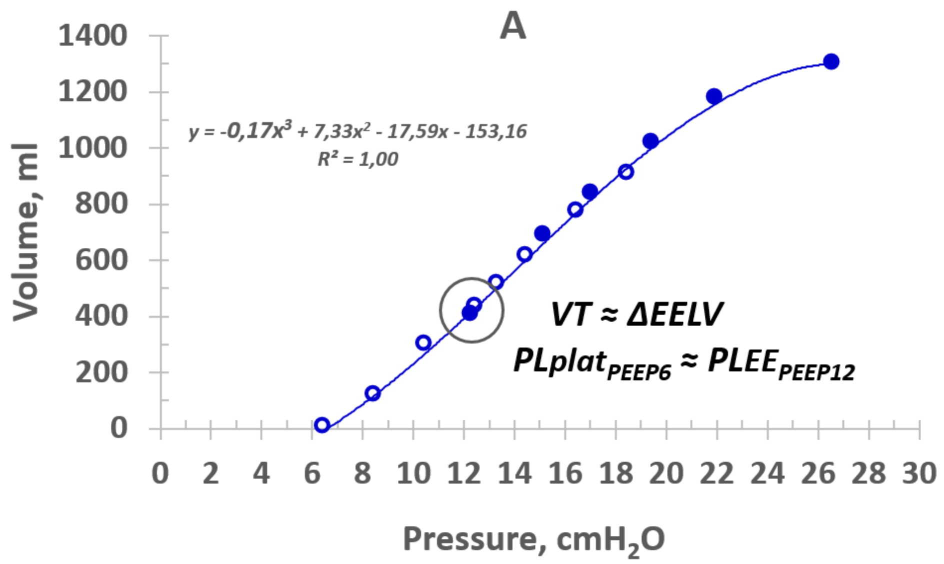

Fig. 3. End-expiratory (blue circles,EE) and end-inspiratory (EI, filled circles) transpulmonary P/V points during 6-step PEEP trial in patient A. Note that EE and EI P/V points are aligned on a single common lung P/V curve. Also note that EE P/V point at PEEP 12 cmH_2O almost coincides with EI P/V point at PEPP 6 cmH_2O as tidal volume is almost the same as ΔEELV between PEEP 6 and 12 cmH_2O.

Analysing according to the Lung Barometry concept (the PEEP step method, PSM)

In figure 1 of the Mojoi et al study, slow inflation P/V curves from each PEEP level are depicted. I have chosen to identify, by manual plotting, the PAWplat and PLplat at each PEEP level for a tidal volume of 400 ml in A patient, 300 ml in the B patient and 200 ml in the C patient. By this plotting procedure, a PAW, PL and volume table was established which formed the basis for calculation of the lung P/V curve from end-expiration at baseline PEEP to end-inspiratio at h highest PEEP level of the PEEP trial (Fig. 4, table 1).

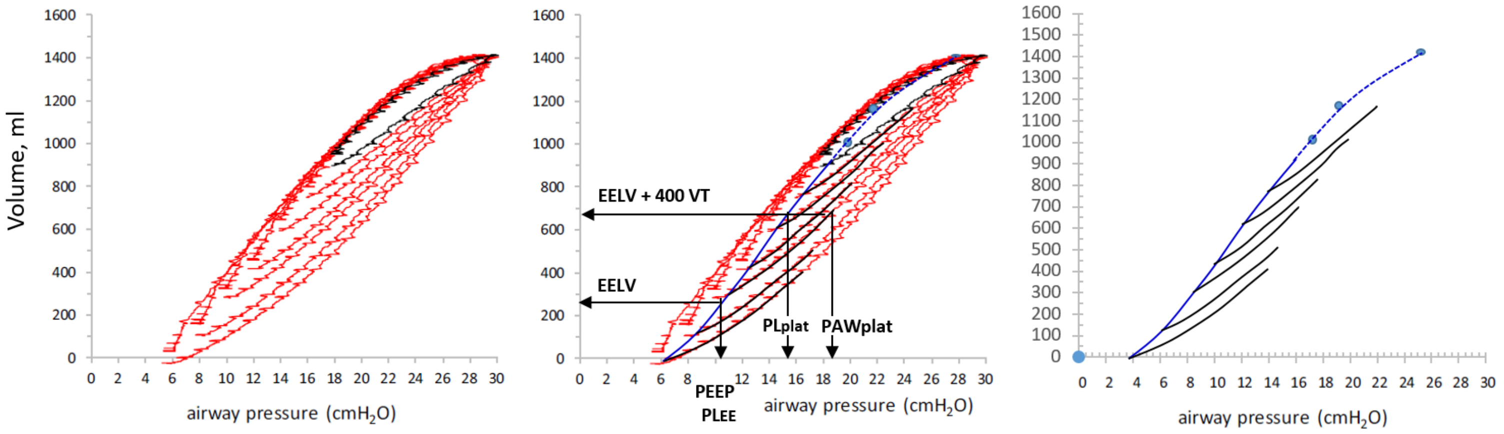

*Fig. 4. **Left panel:** Mojoi original figure of PEEP trial [8]. **Mid panel:** End-expiratory airway (= PEEP) P/V points connected to lung P/V curve (blue line). At each PEEP level a 400 ml tidal airway P/V curves are depicted (black curves). Black horisontal and vertical arrows show how EELV, EELV + tidal volume, PEEP, PLplat and PAWplat were identified by plotting. **Right panel:** Lung P/V curve (blue) and 400 ml tidal airway P/V curves (black).*

	PAW	PL	VOL
EE	6,5	6,5	0
EI	16,6	12,3	400
EE	8,5	8,5	105
EI	17,2	13,4	505
EE	10,5	10,5	290
EI	18,6	15,2	690
EE	12,5	12,5	430
EI	20,1	17,1	830
EE	14,5	14,5	610
EI	22,2	19,5	1010
EE	16,5	16,5	770
EI	24,4	22,0	1170
EE	18,5	18,5	910
EI	29,0	26,7	1300

Table 1. Airway, ranspulmonary pressure (PAW, PL) and volume (VOL)table of PEEP trial with 400 ml tidal volume in patient A of Mojoli et al study.

The lung P/V curve is derived from 6 PEEP steps of the original Mojoli PEEP trial (Fig. 4) and the procedure is more than 20 minutes long. However, the PEEP step method makes it possible to obtain an almost identical lung P/V curve by a 2-PEEP step procedure in around 3 minutes (Fig. 5).

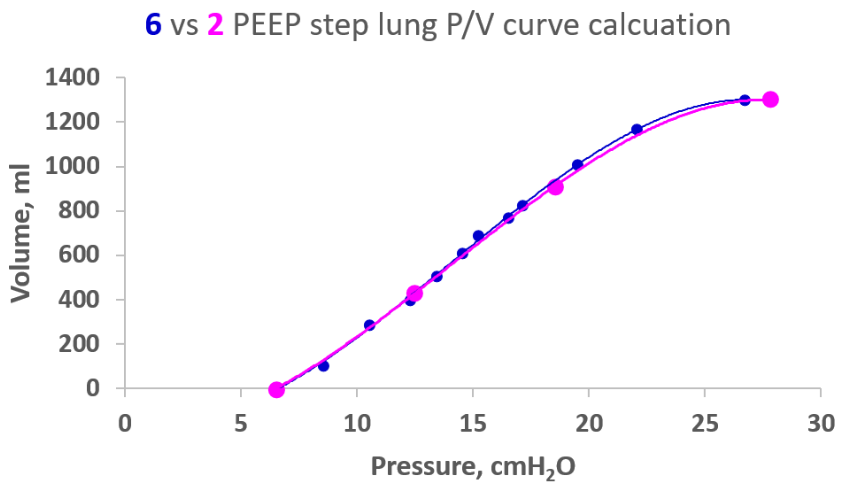

Fig. 5. Lung P/V curve plotted from the 6 PEEP step procedure of Mojoli study (blue P/V points and blue line) and from a two PEEP step procedure by the PEEP step method (4 magenta transpulmonary P/V points and magenta lung P/V curve) [9]

1. Pelosi P, Cereda M, Foti G, Giacomini M, Pesenti A: **Alterations of lung and chest wall mechanics in patients with acute lung injury: effects of positive end-expiratory pressure**. *American journal of respiratory and critical care medicine* 1995, **152**(2):531-537.
2. Gattinoni L, Pelosi P, Suter PM, Pedoto A, Vercesi P, Lissoni A: **Acute respiratory distress syndrome caused by pulmonary and extrapulmonary disease. Different syndromes?** *American journal of respiratory and critical care medicine* 1998, **158**(1):3-11.
3. Garnero A, Tuxen D, Ducros L, Demory D, Donati SY, Durand-Gasselin J, Cooper J, Hodgson C, Arnal JM: **Non-invasive assessment of lung elastance in patients with acute respiratory distress syndrome**. *Minerva anestesiologica* 2015, **81**(10):1096-1104.
4. Lundin S, Grivans C, Stenqvist O: **Transpulmonary pressure and lung elastance can be estimated by a PEEP-step manoeuvre**. *Acta anaesthesiologica Scandinavica* 2015, **59**(2):185-196.
5. Persson P, Stenqvist O, Lundin S: **Evaluation of lung and chest wall mechanics during anaesthesia using the PEEP-step method**. *British journal of anaesthesia* 2018, **120**(4):860-867.
6. Grivans C, Lundin S, Stenqvist O, Lindgren S: **Positive end-expiratory pressure-induced changes in end-expiratory lung volume measured by spirometry and electric impedance tomography**. *Acta anaesthesiologica Scandinavica* 2011, **55**(9):1068-1077.
7. Stenqvist O: **Transpulmonary driving pressure, without esophageal pressure measurements, instead of airway driving pressure**. *Intensive care medicine* 2020, **46**(11):2113-2114.
8. Mojoli F, Pozzi M, Arisi E, Mongodi S, Orlando A, Maggio G, Capra Marzani F, Brochard L: **Tidal lung hysteresis to interpret PEEP-induced changes in compliance in ARDS patients**. *Critical care* 2023, **27**(1):233.
9. Grivans C, Stenqvist O: **Gas distribution by EIT during PEEP inflation: PEEP response and optimal PEEP with lowest trans-pulmonary driving pressure can be determined without esophageal pressure during a rapid PEEP trial in patients with acute respiratory failure**. *Physiological measurement* 2022, **43**(11).
